# Higher Cerebral Small Vessel Disease Burden in Patients With Small Intracerebral Hemorrhage

**DOI:** 10.3389/fnins.2022.888198

**Published:** 2022-05-12

**Authors:** Zi-Jie Wang, Rui Zhao, Xiao Hu, Wen-Song Yang, Lan Deng, Xin-Ni Lv, Zuo-Qiao Li, Jing Cheng, Ming-Jun Pu, Zhou-Ping Tang, Guo-Feng Wu, Li-Bo Zhao, Peng Xie, Qi Li

**Affiliations:** ^1^Department of Neurology, The First Affiliated Hospital of Chongqing Medical University, Chongqing, China; ^2^Department of Neurology, Yongchuan Hospital of Chongqing Medical University, Chongqing, China; ^3^National Health Commission (NHC) Key Laboratory of Diagnosis and Treatment on Brain Functional Diseases, The First Affiliated Hospital of Chongqing Medical University, Chongqing, China; ^4^Department of Neurology, Tongji Hospital, Tongji Medical College, Huazhong University of Science and Technology, Wuhan, China; ^5^Emergency Department, The Affiliated Hospital of Guizhou Medical University, Guiyang, China; ^6^Department of Neurology, Harvard Medical School, Massachusetts General Hospital, Boston, MA, United States

**Keywords:** intracerebral hemorrhage, cerebral small vessel disease, computed tomography, magnetic resonance imaging, neuroimaging, stroke

## Abstract

**Objective:**

To investigate the association between cerebral small vessel disease (SVD) and hematoma volume in primary intracerebral hemorrhage (ICH).

**Methods:**

Patients from a prospective ICH cohort were enrolled. Admission and follow-up CT scan within 72 h after onset were reviewed to calculate the final hematoma volume. We evaluated cortical superficial siderosis and the global SVD score, including white matter hyperintensities, lacunes, enlarged perivascular space, and cerebral microbleeds on MRI. We conducted the multivariate logistic regression analyses to explore the association between SVD markers and small ICH, as well as hematoma volume. Hematoma location was stratified into lobar and non-lobar for subgroup analysis.

**Results:**

A total of 187 patients with primary ICH (mean age 62.4 ± 13.4 years, 67.9% male) were enrolled. 94 (50.2%) patients had small ICH. The multivariate logistic regression analysis showed an association between global SVD score and small ICH [adjusted odds ratio (aOR) 1.27, 95% CI 1.03–1.57, *p* = 0.027] and a trend of higher global SVD score towards non-lobar small ICH (aOR 1.23, 95% CI 0.95–1.58, *p* = 0.122). In the multivariate linear regression analysis, global SVD score was inversely related to hematoma volume of all ICH (β = −0.084, 95% CI −0.142 to −0.025, *p* = 0.005) and non-lobar ICH (β = −0.112, 95% CI −0.186 to −0.037, *p* = 0.004). Lacune (β = −0.245, 95% CI −0.487 to −0.004, *p* = 0.046) was associated with lower non-lobar ICH volume.

**Conclusion:**

Global SVD score is associated with small ICH and inversely correlated with hematoma volume. This finding predominantly exists in non-lobar ICH.

## Introduction

Spontaneous intracerebral hemorrhage (ICH) is a mortal subtype of stroke affecting more than 2 million people worldwide (Qureshi et al., 2009; Cordonnier et al., 2018). Short-term case fatality rate is estimated to exceed 40%, whereas approximately 70% are demised after 5 years (van Asch et al., 2010; Poon et al., 2014). ICH is the most devastating manifestation of cerebral small vessel disease (SVD) (Pantoni, 2010). SVD has been considered as the underlying cause of more than 80% of ICH cases (Rodrigues et al., 2021).

Large hematoma volume and early hematoma expansion are the most robust predictors of neurological deterioration and unfavorable outcomes and, meanwhile, the crucial modifiable targets (Davis et al., 2006). Previously, we have demonstrated that non-contrast CT markers are reliable to identify ICH at high risk of expansion (Li et al., 2015, 2016, 2017, 2019a). Recently, we proposed the definition of small and benign ICH as a subgroup at low risk of hematoma expansion and higher likelihood of functional independence (Li et al., 2019b). Intensive care and aggressive antiexpansion therapies may not be necessary for this subgroup.

ICH location is associated with different underlying types of SVD. Hemorrhage in deep and infratentorial regions is attributed to hypertensive arteriolosclerosis, whereas lobar ICH is related to cerebral amyloid angiopathy (CAA) (Charidimou et al., 2012). SVD markers on MRI such as white matter hyperintensity (WMH), lacune, perivascular space (PVS), and cerebral microbleed (CMB) well represent the underlying microangiopathic damage. Although it is hypothesized that SVD may promote extensive bleeding in the acute phase, several studies discussing the effect of SVD to hematoma volume and expansion showed mixed results (Lou et al., 2010; Boulouis et al., 2016; Sykora et al., 2017; Uniken Venema et al., 2020; Xu et al., 2021). One study reported greater white matter damage in larger ICH (Lou et al., 2010), while others found insignificant or borderline association between WMH and ICH volume (Boulouis et al., 2016; Sykora et al., 2017; Uniken Venema et al., 2020). There are limited data illustrating the association between the overall SVD burden and ICH volume.

In this study, we aim to investigate whether global SVD score is associated with small ICH. We further conducted a subgroup analysis by stratifying the hematoma location into lobar and non-lobar ICH.

## Materials and Methods

### Study Design and Participants

We consecutively collected the clinical and imaging information from an ongoing prospective cohort of primary ICH admitted to the First Affiliated Hospital of Chongqing Medical University from January 2015 to November 2021. Patients aged > 18 years were included, if they had: (1) at least one CT scan within 72 h after symptomatic onset and (2) brain MRI scan, including blood-sensitive susceptibility-weighted imaging (SWI) within 30 days from onset. Patients were excluded, if they had: (1) secondary ICH attributable to structural lesions (e.g., arteriovenous malformation, intracranial aneurysm, Moyamoya disease, and cavernous angioma), medication-related anticoagulative disorder, trauma, and hemorrhagic transformation from ischemic stroke; (2) multiple ICH; (3) primary intraventricular hemorrhage; and (4) hematoma evacuation before MRI.

### Data Collection

The baseline demographic and clinical characteristics, including age, sex, medical history, and vascular risk factors, were collected by interview and review of electronic medical records. Blood glucose and international normalized ratio (INR) were tested immediately at admission. Serum total cholesterol (TC), triglyceride (TG), high-density lipoprotein cholesterol (HDL-c), and low-density lipoprotein cholesterol (LDL-c) were obtained within 24 h after admission.

### Image Acquisition and Analysis

We collected all the CT and MR images as Digital Imaging and Communications in Medicine (DICOM) format for further review.

All the CT images were independently evaluated by two trained neurologists blinded to clinical information. ICH location was dichotomized into lobar (i.e., cortical and subcortical white matter) and non-lobar (i.e., basal ganglia, thalamus, cerebellum, and brainstem) (Charidimou et al., 2017b). The multiple ICH was defined as coexistence of more than two non-contiguous parenchymal hematoma on baseline CT scan (Wu et al., 2017). ICH volume on CT was measured using ABC/2 method (Kothari et al., 1996). The final hematoma volume was defined as the largest hematoma volume on brain CT scan within 72 h from ictus. Small ICH was assessed based on the final hematoma volume fulfilling the following criteria: (1) volume < 3 ml for brainstem ICH; (2) volume < 5 ml for cerebellar ICH; (3) volume < 10 ml for basal ganglia and thalamic ICH; and (4) volume < 15 ml for lobar ICH (Li et al., 2019b).

All the MR images were independently reviewed by two trained neurologists blinded to clinical data according to the STandards for ReportIng Vascular changes on nEuroimaging (STRIVE) criteria (Wardlaw et al., 2013). Deep and periventricular white matter hyperintensities (WMHs) were visually assessed using the Fazekas scale (Fazekas et al., 1987). Dilated perivascular space (PVS) was visually assessed on T2-weighted images (Doubal et al., 2010). Lacune was defined as round or ovoid cavities of low signal with or without light rim on fluid-attenuated inversion recovery (FLAIR) and corresponding cerebrospinal fluid (CSF) signal on T2-weighted images without hypointense signal on susceptibility-weighted imaging (SWI). Cerebral microbleed (CMB) was defined as round or ovoid lesion of homogeneous low signal on SWI (Greenberg et al., 2009b). CMB location was classified as deep (affecting basal ganglia, thalamus, and brainstem) and lobar (affecting cortex and subcortical white matter) and further categorized as strictly and non-strictly lobar CMB. Cortical superficial siderosis (cSS) was defined as curvilinear intrasulcal hypointensities on SWI (Charidimou et al., 2015). Any discrepancies were solved by further review and discussion.

### Rating Scale of Overall Small Vessel Disease Severity

The SVD severity was assessed by global SVD score, a validated MRI-based ordinal rating scale from 0 to 6 points (Lau et al., 2017; Pasi et al., 2021). One point was allocated for: (1) moderate WMH (total Fazekas score 3–4); (2) moderate-to-severe basal ganglia-dilated perivascular space (BG-PVS) (> 20); (3) presence of one or more lacunes; and (4) 1–4 any CMBs. Two points were allocated for: (1) severe WMH (total Fazekas score 5–6) and (2) ≥ 5 any CMBs. We dichotomized the SVD severity as mild (global SVD score < 3 points) and severe (global SVD score ≥ 3 points) (Pasi et al., 2021).

### Statistical Analysis

In the univariate analysis, we compared the clinical and neuroimaging characteristics between patients with small and non-small ICH. Categorical variables were presented as number and percentages. Continuous variables were presented as mean (SD) or median [interquartile range (IQR)] depending on distribution. We compared continuous and ordinal categorical variables with the Student’s *t*-test or the Mann–Whitney *U*-test and categorical variables with the chi-squared test or the Fisher’s exact test, as appropriate. We investigated the association between SVD and small ICH in the multivariate logistic regression analysis by entering prespecified covariates and variables with *p* < 0.1 in the univariate analysis. We further conducted the multivariate linear regression analysis, including log-transformed final hematoma and covariates from the univariate analysis. The model 1 included individual SVD markers and the model 2 included the global SVD score. cSS is a well-established imaging marker of large hematoma volume, we included it into the model despite of no significance in the univariate analysis. In subgroup analysis, we investigated the effect of SVD to small ICH in lobar and non-lobar ICH separately. A *p* < 0.05 was considered as statistically significant. All the analyses were performed with the SPSS for Windows, version 26.

## Results

**FIGURE 1:**
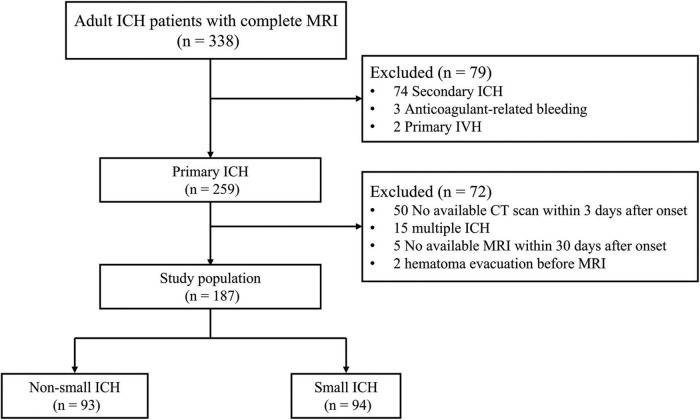
Study flowchart.

A total of 338 patients who met the inclusion criteria were admitted to our hospital between this study period. A total of 151 patients were excluded for meeting one or more of exclusion criteria. The flowchart of selecting patients eligible for analysis is given in Figure 1. Finally, 187 patients (mean age 62.4 ± 13.4 years, 67.9% male) were enrolled in this analysis (Table 1). Of them, 57 (30.5%) patients had lobar ICH. The median time from ICH onset to MRI was 6 days [interquartile range (IQR) 4–9 days].

**TABLE 1:** Baseline clinical and imaging characteristics of the study population.

	Study population (*n* = 187)
**Clinical characteristics**	
Age, mean (*SD*), year	62.4 (13.4)
Male, *n* (%)	127 (67.9)
Hypertension, *n* (%)	160 (85.6)
Diabetes, *n* (%)	42 (22.5)
Dyslipidemia, *n* (%)	47 (25.1)
Previous IS, *n* (%)	11 (5.9)
Previous ICH, *n* (%)	16 (8.6)
Current smoker, *n* (%)	70 (37.4)
Alcohol use, *n* (%)	41 (21.9)
**Imaging characteristics**	
Time from onset to MRI, median (IQR), d	6 (4–9)
Lobar ICH, *n* (%)	57 (30.5)
Baseline hematoma volume, median (IQR), mL	9.1 (2.5–19.0)
Final hematoma volume, median (IQR), mL	10.1 (2.7–20.4)
Baseline intraventricular hemorrhage, *n* (%)	22 (11.8)
WMH Fazekas score, median (IQR)	3 (2–5)
Presence of lacune, *n* (%)	70 (37.4)
Severe CSO-PVS, *n* (%)	39 (20.9)
Severe BG-PVS, *n* (%)	11 (5.9)
Presence of CMB, *n* (%)	150 (80.2)
Total CMB count, median (IQR)	3 (1–15)
CMB ≥ 5, *n* (%)	83 (44.4)
Presence of cSS, *n* (%)	18 (9.6)
Global SVD score, median (IQR)	2 (1–4)

*BG, basal ganglia; CSO, centrum semiovale; cSS, cortical superficial siderosis; CMB, cerebral microbleed; CHD, coronary heart disease; ICH, intracerebral hemorrhage; IS, ischemic stroke; IQR, interquartile range; MRI, magnetic resonance imaging; SD, standard deviation; SVD, small vessel disease; PVS, perivascular space; WMH, white matter hyperintensity.*

**FIGURE 2:**
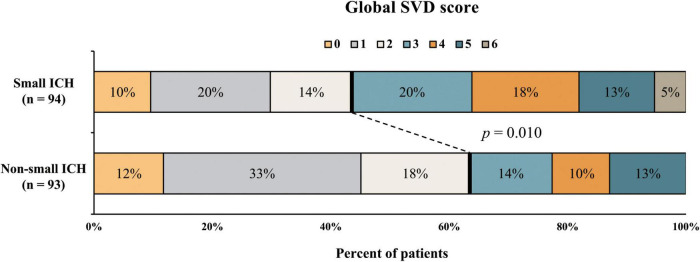
Distribution of small vessel disease (SVD) severity in patients with small and non-small intracerebral hemorrhage (ICH).

Among the study population, small ICH was presented in 94 (50.2%) patients. The univariate analysis of patients with small and non-small ICH is given in Table 2. The median baseline and final hematoma volume were smaller in small ICH as compared with non-small ICH (*p* < 0.001). Hypertension was numerically more prevalent in small ICH (90.4 vs. 80.6%, *p* = 0.090). Other demographic, clinical, and laboratory characteristics were comparable between patients with small and non-small ICH (all *p* > 0.10). In the univariate analysis, patients with small ICH were more likely to have higher WMH burden (*p* = 0.058), lacune (*p* = 0.027), CMB ≥ 5 (*p* = 0.022), and higher global SVD score (*p* = 0.014), whereas lobar ICH (*p* = 0.004) was more frequent in patients with non-small ICH. The prevalence of cSS was higher in patients with non-small ICH, albeit no statistical significance. Severe SVD was presented in 53 of 94 patients with small ICH (56%) and 34 of 93 patients with non-small ICH (37%, *p* = 0.010, Figure 2).

**TABLE 2:** Baseline clinical and imaging characteristics of patients with small and non-small ICH.

	Small ICH (*n* = 94)	Non-small ICH (*n* = 93)	*p*-value
**Clinical characteristics**			
Age, mean (*SD*), year	63.3 (14.3)	61.5 (12.5)	0.365
Male, *n* (%)	61 (63.5)	66 (72.5)	0.246
Hypertension, *n* (%)	85 (90.4)	75 (80.6)	0.090
Diabetes, *n* (%)	19 (20.2)	23 (24.7)	0.572
Dyslipidemia, *n* (%)	22 (23.4)	25 (26.9)	0.704
Previous IS, *n* (%)	4 (4.3)	7 (7.5)	0.372
Previous ICH, *n* (%)	6 (6.4)	10 (10.8)	0.420
Current smoker, *n* (%)	37 (39.4)	33 (35.5)	0.692
Alcohol use, *n* (%)	17 (18.1)	24 (25.8)	0.272
**Laboratory characteristics**			
Admission blood glucose, median (IQR), mmol/L	7.8 (6.1–9.1)	7.2 (6.0–8.6)	0.465
TC, mean (SD), mmol/L	4.57 (0.99)	4.48 (0.92)	0.579
TG, median (IQR), mmol/L	1.12 (0.83–1.81)	1.36 (0.80–1.65)	0.672
HDL-c, mean (SD), mmol/L	1.28 (0.36)	1.30 (0.38)	0.758
LDL-c, mean (SD), mmol/L	2.85 (0.87)	2.77 (0.82)	0.519
International normalized ratio, mean (SD)	1.00 (0.08)	1.00 (0.06)	0.768
**Imaging characteristics**			
Lobar ICH, *n* (%)	19 (20.2)	38 (40.9)	0.004
Baseline hematoma volume, median (IQR), mL	2.5 (0.9–5.6)	19.0 (14.1–29.7)	<0.001
Final hematoma volume, median (IQR), mL	2.7 (0.9–6.3)	20.6 (15.3–31.7)	<0.001
Baseline intraventricular hemorrhage, *n* (%)	10 (10.6)	12 (12.9)	0.800
WMH Fazekas score, median (IQR)	3 (2–5)	2 (2–4)	0.058
Presence of lacune, *n* (%)	43 (45.7)	27 (29.0)	0.027
Severe CSO-PVS, *n* (%)	21 (22.3)	18 (19.4)	0.747
Severe BG-PVS, *n* (%)	5 (5.3)	6 (6.5)	0.767
Presence of CMB, *n* (%)	78 (83.0)	72 (77.4)	0.441
Strictly lobar CMB, *n* (%)	9 (9.6)	14 (15.1)	0.359
Non-strictly lobar CMB, *n* (%)	69 (73.4)	58 (62.4)	0.144
Total CMB count, median (IQR)	5 (1–20)	3 (1–8)	0.010
CMB ≥ 5, *n* (%)	50 (53.2)	33 (35.5)	0.022
Presence of cSS, *n* (%)	5 (5.3)	13 (14.0)	0.079
Global SVD score, median (IQR)	3 (1–4)	2 (1–3)	0.014

*BG, basal ganglia; CSO, centrum semiovale; cSS, cortical superficial siderosis; CMB, cerebral microbleed; ICH, intracerebral hemorrhage; IS, ischemic stroke; IQR, interquartile range; SD, standard deviation; SVD, small vessel disease; TC, total cholesterol; TG, triglyceride; HDL-c, high-density lipoprotein cholesterol; LDL-c, low-density lipoprotein cholesterol; PVS, perivascular space; WMH, white matter hyperintensity.*

In the multivariate logistic regression analysis (Table 3), after adjusting for age, male sex, and hypertension, no individual SVD marker was independently associated with small ICH. Global SVD score [adjusted odds ratio (aOR) 1.27, 95% confidence interval (CI) 1.03–1.57, *p* = 0.027] was correlated with small ICH. By contrast, lobar ICH was correlated with non-small ICH. In subgroup analysis of hematoma location, we observed only a trend of global SVD score (aOR 1.23, 95% CI 0.95–1.58, *p* = 0.123) toward increasing odds of small ICH in patients with non-lobar ICH.

**TABLE 3:** The multivariate logistic regression analysis of variables associated with small ICH adjusted for age and male sex.

	Total cohort			Lobar ICH			Non-lobar ICH		
	aOR	95% CI	*p*-value	aOR	95% CI	*p*-value	aOR	95% CI	*p*-value
Model 1: individual SVD markers*									
Hypertension	1.38	0.53–3.60	0.511	1.70	0.28–10.32	0.567	1.42	0.43–4.76	0.567
Lobar ICH	0.34	0.16–0.73	0.006	–	–	–	–	–	–
WMH Fazekas score	1.06	0.85–1.33	0.606	1.12	0.76–1.64	0.574	1.05	0.79–1.40	0.743
Presence of lacune	1.81	0.89–3.68	0.099	1.01	0.21–4.85	0.989	2.08	0.91–4.78	0.085
CMB ≥ 5	1.62	0.77–3.41	0.205	4.18	0.79–22.25	0.094	1.36	0.56–3.28	0.495
Presence of cSS	0.53	0.14–2.03	0.356	0.53	0.08–2.32	0.325	–	–	1.00
Model 2: global SVD score*									
Hypertension	1.54	0.60–3.94	0.370	1.80	0.32–9.99	0.503	1.60	0.50–5.16	0.431
Lobar ICH	0.36	0.17–0.76	0.008	–	–	–	–	–	–
Presence of cSS	0.55	0.15–2.03	0.372	0.41	0.08–2.03	0.410	–	–	1.00
Global SVD score	1.27	1.03–1.57	0.027	1.44	0.94–2.20	0.094	1.23	0.95–1.58	0.122

* Adjusted for age and male sex. aOR, adjusted odds ratio; CI, confidence interval; cSS, cortical superficial siderosis; CMB, cerebral microbleed; ICH, intracerebral hemorrhage; SVD, small vessel disease; WMH, white matter hyperintensity.

**FIGURE 3:**
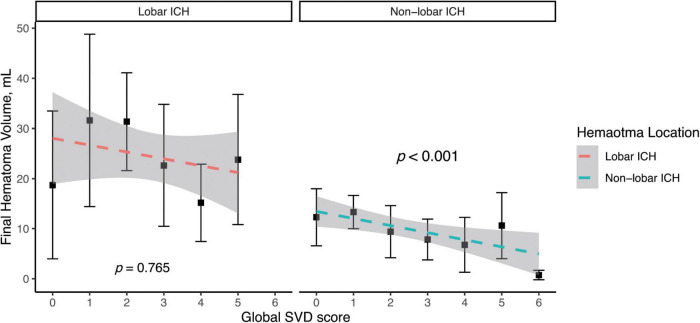
Simple linear regression model of log-transformed hematoma volume and global SVD score in lobar and non-lobar ICH. The dependent variable was base-10 logarithm of hematoma volume. Log of hematoma volume in non-lobar ICH = -0.133 × Global SVD score + 0.993, *p* < 0.001. Log of hematoma volume in lobar ICH = -0.013 × Global SVD score + 1.226, *p* = 0.765.

Figure 3 shows a highly inverse relationship between SVD burden and final hematoma volume in non-lobar (*p* < 0.001) rather than lobar ICH in a simple linear regression model. In the multivariate linear regression analysis (Table 4), there was a significant association of higher global SVD score and lower log-transformed hematoma volume (β = −0.084, 95% CI −0.142 to −0.025, *p* < 0.001) in the whole study and in subgroup of patients with non-lobar ICH (β = −0.112, 95% CI −0.186 to −0.037, *p* = 0.004) after adjusting for age, male sex, and hypertension. For individual SVD markers, lacune (β = −0.245, 95% CI −0.487 to −0.004, *p* = 0.046) was the only SVD marker independently associated with lower non-lobar ICH volume.

**TABLE 4:** The multivariate linear regression analysis of associations between SVD markers and hematoma volume adjusted for age and male sex.

	Total cohort			Lobar ICH			Non-lobar ICH		
	β	95% CI	*p*-value	β	95% CI	*p*-value	β	95% CI	*p*-value
Model 1: individual SVD markers*									
Hypertension	−0.075	−0.339 to 0.188	0.573	−0.101	−0.479 to 0.276	0.592	−0.168	−0.311 to 0.159	0.358
Lobar ICH	0.482	0.268 to 0.696	<0.001	–	–	–	–	–	–
WMH Fazekas score	−0.014	−0.076 to 0.047	0.647	−0.025	−0.116 to 0.066	0.585	−0.007	−0.091 to −0.077	0.867
Presence of lacune	−0.173	−0.372 to 0.027	0.090	0.002	−0.370 to 0.375	0.990	−0.245	−0.487 to −0.004	0.046
CMB ≥ 5	−0.136	−0.347 to −0.074	0.203	−0.039	−0.404 to 0.325	0.829	−0.212	−0.473 to 0.049	0.110
Presence of cSS	0.289	−0.059 to −0.637	0.102	0.317	−0.049 to 0.683	0.088	0.127	−1.127 to 1.381	0.841
Model 2: global SVD score*									
Hypertension	−0.089	−0.349 to 0.170	0.498	−0.098	−0.466 to 0.270	0.595	−0.187	−0.539 to 5.16	0.431
Lobar ICH	0.477	0.265 to 0.690	<0.001	–	–	–	–	–	–
Presence of cSS	0.274	−0.070 to 0.618	0.117	0.332	−0.017 to −0.680	0.062	−0.031	−1.270 to 1.209	0.961
Global SVD score	−0.084	−0.142 to −0.025	0.005	−0.029	−0.122 to 0.065	0.540	−0.112	−0.186 to −0.037	0.004

* Adjusted for age and male sex. Dependent variable was base-10 log-transformed hematoma volume. β, regression coefficient; CI, confidence interval; cSS, cortical superficial siderosis; CMB, cerebral microbleed; ICH, intracerebral hemorrhage; SVD, small vessel disease; WMH, white matter hyperintensity.

To investigate whether the correlation of global SVD burden with small ICH remains consistent according to history of hypertension or ICH location, we further analyzed any potential differences of association between SVD burden and small ICH by hypertension and hematoma location. There was no significant interaction between hypertension and SVD burden (*p* for interaction = 0.195) or between ICH location and SVD burden (*p* for interaction = 0.878, Table 5).

**TABLE 5:** Associations between global SVD score and odds of small ICH by presence of hypertension and hematoma location.

	aOR	95% CI	*p*-value	*p* for interaction
Presence of hypertension*				
With hypertension	1.35	1.08–1.70	0.010	0.195
Without hypertension	0.54	0.23–1.30	0.168	
**Hematoma location^†^**				
Lobar ICH	1.44	0.94–2.19	0.094	0.878
Non-lobar ICH	1.22	0.95–1.58	0.122	

*Adjusted for age, male sex, lobar ICH, and presence of cSS. **^†^** Adjusted for age, male sex, hypertension, and presence of cSS. aOR, adjusted odds ratio; CI, confidence interval; cSS, cortical superficial siderosis; ICH, intracerebral hemorrhage; SVD, small vessel disease; WMH, white matter hyperintensity.

## Discussion

The main finding of this study is that higher SVD burden is associated with small ICH. Data indicated that this association persisted predominantly in non-lobar ICH. With regard to individual SVD markers, only lacune is correlated with lower ICH volume.

Accumulating evidence suggests that large hematoma volume robustly predicts unfavorable outcomes in patients with ICH (Davis et al., 2006). Conversely, smaller ICH volume had lower risk of expansion, neurological worsening, and greater odds of good outcomes (Dowlatshahi et al., 2016). In this study, we defined the criteria of small ICH based on different location and reported that small ICH independently predicts 3-month good outcomes (Li et al., 2019b).

Small vessel disease markers have been demonstrated to predict future ICH, while our data suggesting that they may not lead to severe hemorrhagic event, which is of great interest (Debette et al., 2019). Prior studies investigating microangiopathy and hematoma volume showed inconsistent results. In a retrospective analysis of 79 patients, extensive WMH was associated with greater hematoma volume and early hematoma growth (Lou et al., 2010). Other studies found neutral effect of WMH to hematoma volume (Boulouis et al., 2016; Sykora et al., 2017; Hansen et al., 2020; Uniken Venema et al., 2020). A CT-based study of 2,579 patients with ICH found a borderline opposing association between leukoaraiosis and ICH volume, while MRI markers were not included in this study (Uniken Venema et al., 2020). As a hemorrhagic SVD marker, CMB is associated with elevated risk of ICH in community dwellers (Akoudad et al., 2015), patients with ischemic stroke (Wilson et al., 2019), and those with ICH (Charidimou et al., 2017a). However, there is emerging evidence indicating that CMB may not predict large ICH. A study of 59 patients found significant decreased CMB number in patients with CT angiography spot sign, a hallmark of active blood extravasation (Evans et al., 2010). Another MRI-based study found absence of CMB that was associated with larger ICH volume and no relationship between WMH and hematoma volume in both the deep and lobar ICH (Boulouis et al., 2016). In this study, we found higher global SVD score predisposed to small ICH. In addition, we observed a significant opposing association in the multivariate linear analysis and a marginal correlation between greater SVD burden and non-lobar small ICH in the multivariate logistic regression analysis. Our results corroborate with a recent study, which found an opposing linear relationship between SVD burden and hematoma volume (Xu et al., 2021). With regard to individual SVD markers, WMH, lacune, and severe CMB burden were more frequently observed in small ICH in the univariate analysis, whereas only lacune persisted an association with lower non-lobar ICH volume in the multivariate linear regression analysis. Although there was a trend of increasing CMB number toward small ICH and cSS toward large ICH, SVD markers and global SVD score were not associated with lobar ICH volume in the multivariate logistic regression analysis.

It is highly speculated that coexistence of multiple SVD markers is a sign of cerebrovascular fragility. Non-lobar ICH is related to age and vascular risk factors such as hypertension, diabetes mellitus, dyslipidemia, smoking, and alcohol use (Pantoni, 2010). There is strong evidence that deposition of hyaline material in the thickened tunica media is a major feature of arteriosclerotic changes, which may diminish the risk of active bleeding (Mitchell, 2008). Fisher found that secondary growth surrounding original bleeding site was more likely to be present with vessel without arteriolosclerosis (Fisher, 1971). Source of bleeding in non-lobar ICH has been pathologically proved to be thin-walled aneurysmal dilatation on deep small vessels (Miller Fisher, 2003). In addition, cerebrovascular reactivity (CVR), a parameter of vessel constriction and dilatation ability, was impaired in patients with hypertensive ICH and advanced SVD, which may decrease the risk of progressive bleeding, while the exact impact of reduced CVR to ICH volume remains unknown (Lee et al., 2021). Clinical and experimental studies are needed to validate the potential effect of hemodynamic features to ICH volume. Lobar ICH is CAA related. CMB and cSS may have different clinical and pathological entities (Shoamanesh et al., 2014). An autopsy study of patients with CAA found significantly thicker vessel walls in subjects with high microbleed burden as compared with those with low microbleed burden, which predisposes to protective effects against extensive bleeding (Greenberg et al., 2009a). On the contrary, cSS is a key SVD marker in CAA with high risk of lobar ICH (Charidimou et al., 2019). Neuropathological study of patients with CAA found that cSS was associated with advanced leptomeningeal vessel damage, but reduced cortical amyloid deposition (Charidimou et al., 2020). This study adds evidence to distinct disease entities of CMB and cSS. We observed an increasing CMB number in small ICH and higher prevalence of cSS in non-small ICH, but no significant association between SVD markers and lobar ICH volume was found in the multivariate logistic regression analysis. It is noted that only 57 (30.5%) patients with lobar ICH were included, thus this subgroup may be underrepresented in our analysis.

This study has important clinical implications. First, this study adds evidence to the association between the overall SVD burden and ICH volume. Future studies, including more SVD features such as cerebral microinfarct, may provide more information on the relationship between SVD severity and ICH volume. Second, this study gains insight into the potential role of different SVD markers to hematoma size. Traditional SVD markers, including WMH, lacune, and CMB, may restrict hematoma formation at the acute phase, whereas cSS is prone to active bleeding following superficial small vessel rupture.

There are several limitations. First, this is a single-center study with relatively small sample size, which may attenuate the generalizability. Second, patients with extremely lethal conditions did not receive MRI scan, which may cause selection bias. Third, the global SVD score may not cover the whole spectrum of SVD change. However, the global SVD score included well-recognized SVD markers with stratified severity such as WMH and CMB.

In summary, this study provides better insight into the influence of overall SVD severity to hematoma volume. An inverse correlation between global SVD score and ICH volume was observed. The associations remain significant for non-lobar ICH only. Future studies validate this relationship and exploring the pathophysiological mechanisms is required.

## Data Availability Statement

The data that support the findings of this study are available from the corresponding author on reasonable request by contacting QL, qili_md@126.com.

## Ethics Statement

The studies involving human participants were reviewed and approved by the Ethics Committee of the First Affiliated Hospital of Chongqing Medical University. The patients/participants provided their written informed consent to participate in this study.

## Author Contributions

QL and Z-JW: study concept and design. Z-JW: statistical analysis and writing the manuscript. RZ, XH, W-SY, LD, X-NL, Z-QL, JC, and M-JP: acquisition of data. PX: administrative support. QL: critical revision of the manuscript for intellectual content and obtained funding. All authors: analysis and interpretation of data.

## Conflict of Interest

The authors declare that the research was conducted in the absence of any commercial or financial relationships that could be construed as a potential conflict of interest.

## Publisher’s Note

All claims expressed in this article are solely those of the authors and do not necessarily represent those of their affiliated organizations, or those of the publisher, the editors and the reviewers. Any product that may be evaluated in this article, or claim that may be made by its manufacturer, is not guaranteed or endorsed by the publisher.
